# Full-dose versus reduced-dose comparison of direct oral anticoagulants for extended treatment of venous thromboembolism: a systematic review and meta-analysis of randomized controlled trials

**DOI:** 10.3389/fphar.2025.1708316

**Published:** 2025-12-04

**Authors:** Renshu Zhang, Rui Wang, Yun Li, Wenqi Tian, Dandan Niu, Wencong Cheng, Xiaofang Niu

**Affiliations:** 1 Department of Pharmacy, Heze Hospital Affiliated of Shandong First Medical University, Heze Municipal Hospital, Heze, Shandong, China; 2 Department of Pharmacy, Binzhou People’s Hospital, Binzhou, Shandong, China

**Keywords:** dose, direct oral anticoagulants, venous thromboembolism, extended treatment, randomized controlled trials

## Abstract

**Background:**

For patients requiring extended anticoagulation therapy, clinicians often prescribe reduced-dose direct oral anticoagulants (DOACs) rather than full-dose, which may be related to concerns about the higher bleeding risk associated with full-dose DOACs, despite potentially better efficacy in preventing VTE recurrence. This meta-analysis aims to evaluate the net clinical benefit of reduced-dose DOACs versus full-dose DOACs in extended anticoagulation therapy.

**Methods:**

This study has been registered in the International Prospective Register of Systematic Reviews (PROSPERO identifier: CRD420251089110). A systematic search was conducted in the PubMed, Embase, and Web of Science databases from their inception to 30 June 2025. Data extraction was done independently and in duplicate. A random-effects meta-analysis model was used to report the pooled treatment effects and 95% confidence intervals.

**Results:**

A total of 5 randomized clinical trials were included (8,781 cases). Compared with full-dose DOAC, reduced-dose DOAC did not significantly increase the risk of recurrent VTE or death [(RR, 0.94 (95% CI, 0.68–1.29)), (RR, 0.84 (95% CI, 0.65–1.09))], but significantly reduced the risk of major bleeding/CRNMB (RR, 0.71 (95% CI, 0.61–0.82)). In the analysis of DOAC drugs, the prospective estimates for recurrent VTE were as follows: apixaban, RR, 0.93 (95% CI, 0.63–1.37); rivaroxaban, RR 0.96 (95% CI, 0.54–1.69). The prospective estimates for major bleeding/CRNMB were as follows: apixaban, RR, 0.74 (95% CI, 0.63–0.89); rivaroxaban, RR, 0.63 (95% CI, 0.48–0.84). Most findings were consistent within subgroups.

**Conclusion:**

Reduced-dose DOACs were associated with a significant decrease in the risk of major bleeding/CRNMB compared with full-dose DOACs, but were not associated with a significant increase in the risk of recurrent VTE. These findings support the net clinical benefit of reduced-dose DOACs compared with full-dose DOACs and reinforce adherence with current VTE guidelines.

**Systematic Review Registration:**

Identifier CRD420251089110.

## 1 Introduction

Reduced-dose direct oral anticoagulants (DOACs) are commonly used in clinical practice for extended anticoagulation therapy, which helps prevent recurrent venous thromboembolism (VTE) and reduces bleeding (Stevens et al., 2021; Ortel et al., 2020). However, the net clinical benefit of reduced-dose DOACs compared to full-dose treatment in high-risk VTE patients, such as those with cancer, is not well-defined (Ghorbanzadeh et al., 2025). In these situations, any potential advantages of reduced-dose DOACs must be carefully balanced against the potential increase in recurrent VTE risk.

Whether recurrent VTE and bleeding risk differs between reduced-dose and full-dose DOACs remains unclear. An analysis of the AMPLIFY-EXT (reduced-dose and full-dose apixaban versus placebo for extended treatment of VTE) trial showed no difference between reduced-dose and full-dose apixaban in the prevention of recurrent VTE and even a lower risk of bleeding in extended VTE treatment. However only 15% of the patients were over 75 years old, and few had a weight below 60 kg or moderate to severe renal impairment (Agnelli et al., 2013). Previous meta-analyses have likewise suggested that reduced-dose DOACs may be an attractive option for extended phase anticoagulation after VTE, but the results are not generalizable to groups that were not included or were under-represented in the trials (Vasanthamohan et al., 2018). Clinicians continue to face a difficult challenge in accurately balancing the risks versus benefits of reduced-dose and full-dose DOACs in different populations.

Multiple randomized controlled trials comparing reduced-dose versus full-dose DOACs have recently been completed (McBane et al., 2024; Couturaud et al., 2025; Mahé et al., 2025). By combining events from these and earlier studies, we could estimate the risks of recurrent VTE and bleeding more precisely while possessing sufficient power to analyze key subgroups. We therefore conducted a systematic review and meta-analysis to determine the effects of reduced-dose versus full-dose DOACs on the risks of recurrent VTE and bleeding during extended anticoagulation therapy.

## 2 Methods

Our research was reported in accordance with Preferred Reporting Items for Systematic Reviews and Meta-Analyses (PRISMA) (Page et al., 2021). This study has been registered in the International Prospective Register of Systematic Reviews (PROSPERO identifier: CRD420251089110).

### 2.1 Data sources and search strategy

We identified relevant studies through a systematic literature search in Embase, PubMed, and Web of Science from database inception to 30 June 2025. The searches was not limited by language. We employed a search strategy that combined keywords and MeSH terms related to DOACs and VTE (Supplementary Methods). We also searched the reference lists of included studies and relevant review articles.

### 2.2 Data extraction

We included randomized controlled trials of patients aged 18 years or older with VTE who required extended anticoagulation therapy for at least 6 months, and reported outcomes of recurrent VTE and bleeding events in patients receiving reduced-dose versus full-dose DOACs. Two researchers independently screened each title and abstract according to inclusion and exclusion criteria, with a third researcher conducting further analysis in case of disagreement. Following this screening process, each reviewer assessed the eligibility of full-text articles. We extracted the following data: study characteristics, baseline demographic data of participants, descriptions of the intervention (reduced-dose DOAC) and comparator (full-dose DOAC).

### 2.3 Study outcomes

Our primary outcomes were recurrent VTE and major bleeding/CRNMB. Other outcomes included death from any cause. For major bleeding, we used the definition from the International Society on Thrombosis and Haemostasis, which stipulates one or more of the following: fatal bleeding, symptomatic bleeding in a critical area or organ, or bleeding causing a decrease in hemoglobin level of 20 g/L or more or leading to transfusion of 2 or more units of whole blood or red blood cells (Schulman et al., 2010). We used the Cochrane Collaboration criteria to determine the risk of bias for each included study (Higgins et al., 2011). We then used the Grading of Recommendations, Assessment, Development and Evaluation (GRADE) tool to assess the reporting quality of the primary study results (Guyatt et al., 2011).

### 2.4 Statistical analysis

We conducted meta-analyses separately for each type of DOACs. For each of the meta-analysis, the Mantel-Haenszel method using a random-effects model was employed to calculate the pooled relative risk (RR) and its 95% confidence interval (CI). The I^2^ statistic was used to assess heterogeneity among studies, and a value greater than 50% indicates the presence of substantial heterogeneity (Cochrane, 2024). We conducted *post hoc* subgroup analyses for sex, age, BMI, renal impairment, index VTE, and cancer to explore subgroups that require dose reduction. For these analyses, we performed interaction tests to evaluate possible subgroup effects.

In the RENOVE (high risk of recurrent VTE) trial, patients in the DOACs group received treatment with apixaban or rivaroxaban. Since the proportions of the two were similar, we included them separately in the apixaban and rivaroxaban trial analyses. To assess the stability of the study, we performed a sensitivity analysis using a fixed-effects model instead of a random-effects model.

Risk of bias assessment was performed using RevMan (Version 5.1, Copenhagen: Nordic Cochrane Centre, The Cochrane Collaboration, 2011) software. Data analysis was conducted using R version 4.5.1 software with the package meta. Summary tables related to GRADE were constructed using GRADEpro (Version 3.6. Copyright © 2004–2009 GRADE Working Group). Analyses were 2-tailed, with statistical significance set at a 2-sided P value less than 0.05.

## 3 Result

Through database searching, we identified 1,388 unique citations (Supplementary Figure 1), of which 5 met eligibility criteria (Table 1). Included trials studied patients with extended VTE treatment (VTE, 2 trials (Agnelli et al., 2013; Weitz et al., 2017); cancer-related VTE, 2 trials (McBane et al., 2024; Mahé et al., 2025); high-risk VTE, 1 trial (Couturaud et al., 2025)).

**TABLE 1:** Trial characteristics.

Study, year (Reference)	Trial	Participants, n	Average age, y	Male sex, %	Population	Intervention	Comparator	Follow-up, mo
Agnelli et al. (2013)	AMPLIFY-EXT	1,653	56.5	956 (57.8)	VTE	Apixaban 2.5 mg bid	Apixaban 5 mg bid	12
Weitz et al. (2017)	EINSTEIN CHOICE	2,234	58.4	1,222 (54.7)	VTE	Rivaroxaban 10 mg qd	Rivaroxaban 20 mg qd	12
McBane et al. (2024)	EVE	360	64.0	161 (44.7)	Cancer-related VTE	Apixaban 2.5 mg bid	Apixaban 5 mg bid	12
Couturaud et al. (2025)	RENOVE	2,768	62.7	1797 (64.9)	High risk of recurrent VTE	Apixaban 2.5 mg bid; Rivaroxaban 10 mg qd	Apixaban 5 mg bid; Rivaroxaban 20 mg qd	37.1
Mahé et al. (2025)	API-CAT	1,766	67.5	766 (43.4)	Cancer-related VTE	Apixaban 2.5 mg bid	Apixaban 5 mg bid	12

DOACs, direct oral anticoagulants; mo, month; n, number; y, year; VTE, venous thromboembolism.

Among the 8,781 participants, the average age across the 5 trials was 61.82 years, and trial male population were 55.8% on average. The mean follow-up time across trials was 17 months. All trials reported on study outcomes during the entire follow-up duration, and all reported on the main outcomes of recurrent thromboembolism and major bleeding/CRNMB. Major bleeding was defined per International Society for Thrombosis and Haemostasis criteria for 5 trials.

For the reduced-dose DOACs group, trials used apixaban (2.5 mg twice daily, 4 trials), rivaroxaban (10 mg once daily, 2 trials). For the full-dose DOACs group, trials used apixaban (5 mg twice daily, 4 trials) and rivaroxaban (20 mg once daily, 2 trials). Overall, the risk of bias in the included studies was low (Supplementary Figure 2). Table 2 summarizes our review findings.

**TABLE 2:** Summary of review findings for RCTs comparing reduced-dose versus full-dose DOACs.

Outcome (study type)	No of patients		Effect		Quality of evidence (GRADE)
	Reduced-dose DOACs	Full-dose DOACs	Relative (95% CI)	Absolute	
Recurrent VTE (five RCTs)	73/4,261 (1.7%)	78/4,249 (1.8%)	RR 0.94 (0.68–1.29)	1 fewer per 1,000 (from 6 fewer to 5 more)	MODERATE^a^
Recurrent VTE - apixaban (four RCTs)	48/2,460 (2%)	52/2,459 (2.1%)	RR 0.93 (0.63–1.37)	1 fewer per 1,000 (from 8 fewer to 8 more)	MODERATE^a^
Recurrent VTE - rivaroxaban (two RCTs)	25/1801 (1.4%)	26/1790 (1.5%)	RR 0.96 (0.54–1.69)	1 fewer per 1,000 (from 7 fewer to 10 more)	MODERATE^a^
Major or clinically relevant non-major bleeding (five RCTs)	268/4,266 (6.3%)	383/4,269 (9%)	RR 0.71 (0.61–0.82)	26 fewer per 1,000 (from 16 fewer to 35 fewer)	HIGH
Major or clinically relevant non-major bleeding - apixaban (four RCTs)	193/2,465 (7.8%)	263/2,468 (10.7%)	RR 0.74 (0.63–0.89)	28 fewer per 1,000 (from 12 fewer to 39 fewer)	HIGH
Major or clinically relevant non-major bleeding - rivaroxaban (two RCTs)	75/1,801 (4.2%)	120/1801 (6.7%)	RR 0.63 (0.48–0.84)	25 fewer per 1,000 (from 11 fewer to 35 fewer)	HIGH
Death (four RCTs)	209/3,555 (5.9%)	251/3,573 (7%)	RR 0.84 (0.65–1.09)	11 fewer per 1,000 (from 25 fewer to 6 more)	MODERATE^b^

Cl, confidence interval; DOACs, direct oral anticoagulants; GRADE, grading of recommendation assessment development and evaluation; RCT, randomized controlled trial; RR, risk ratio; VTE, venous thromboembolism.

^a^ Low sample sizes and wide confidence intervals.

^b^ Wide confidence intervals.

### 3.1 Recurrent VTE

**FIGURE 1:**
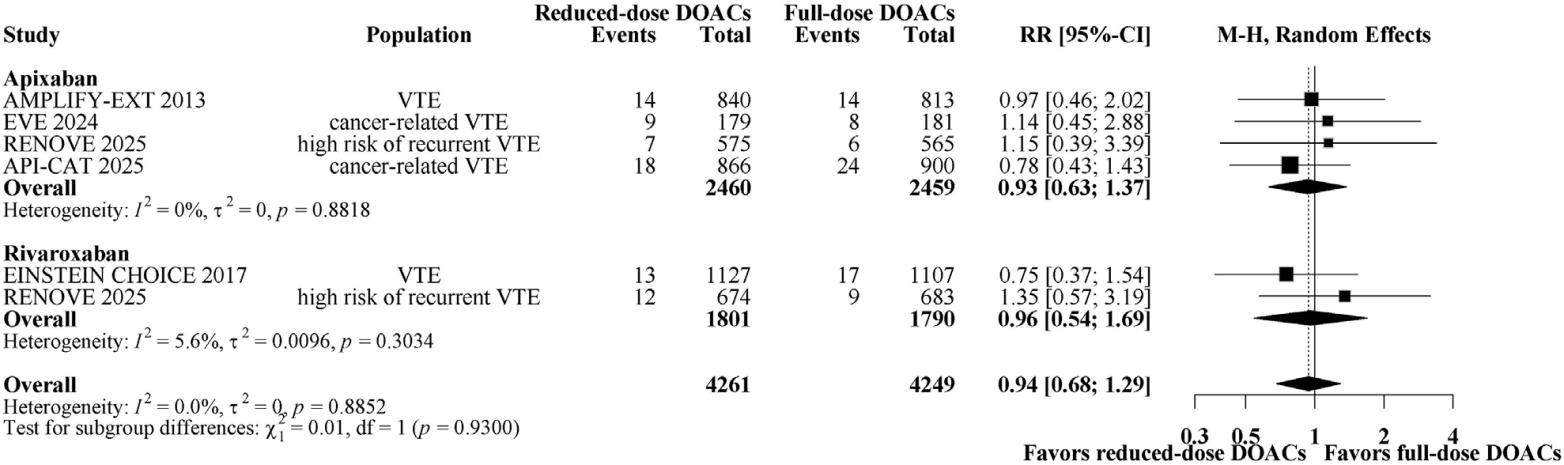
Forest plot of randomized controlled trials comparing reduced-dose DOACs vs. full-dose DOACs for risk for Recurrent VTE.

A total of 151 patients (1.78%) had recurrent VTE. Figure 1 shows a forest plot of trials comparing reduced-dose versus full-dose DOACs on risk for recurrent VTE. Reduced-dose DOACs was not associated with significantly higher risks of recurrent VTE compared with full-dose DOACs (1.7% vs 1.8%; RR, 0.94; 95% CI, 0.68–1.29). There was no heterogeneity among DOACs agents (overall I^2^ = 0%). In the analysis by DOACs agent, the respective estimates for recurrent VTE risk were as follows: apixaban, RR, 0.93 (95% CI, 0.63–1.37); rivaroxaban, RR, 0.96 (95% CI, 0.54–1.69). Sensitivity analysis indicated that the results remain unchanged when using a fixed-effect model (Supplementary Figure 5A). The quality of evidence was moderate for the comparison between reduced-dose and full-dose DOACs, also being graded down because of imprecision (Table 2).

**FIGURE 2:**
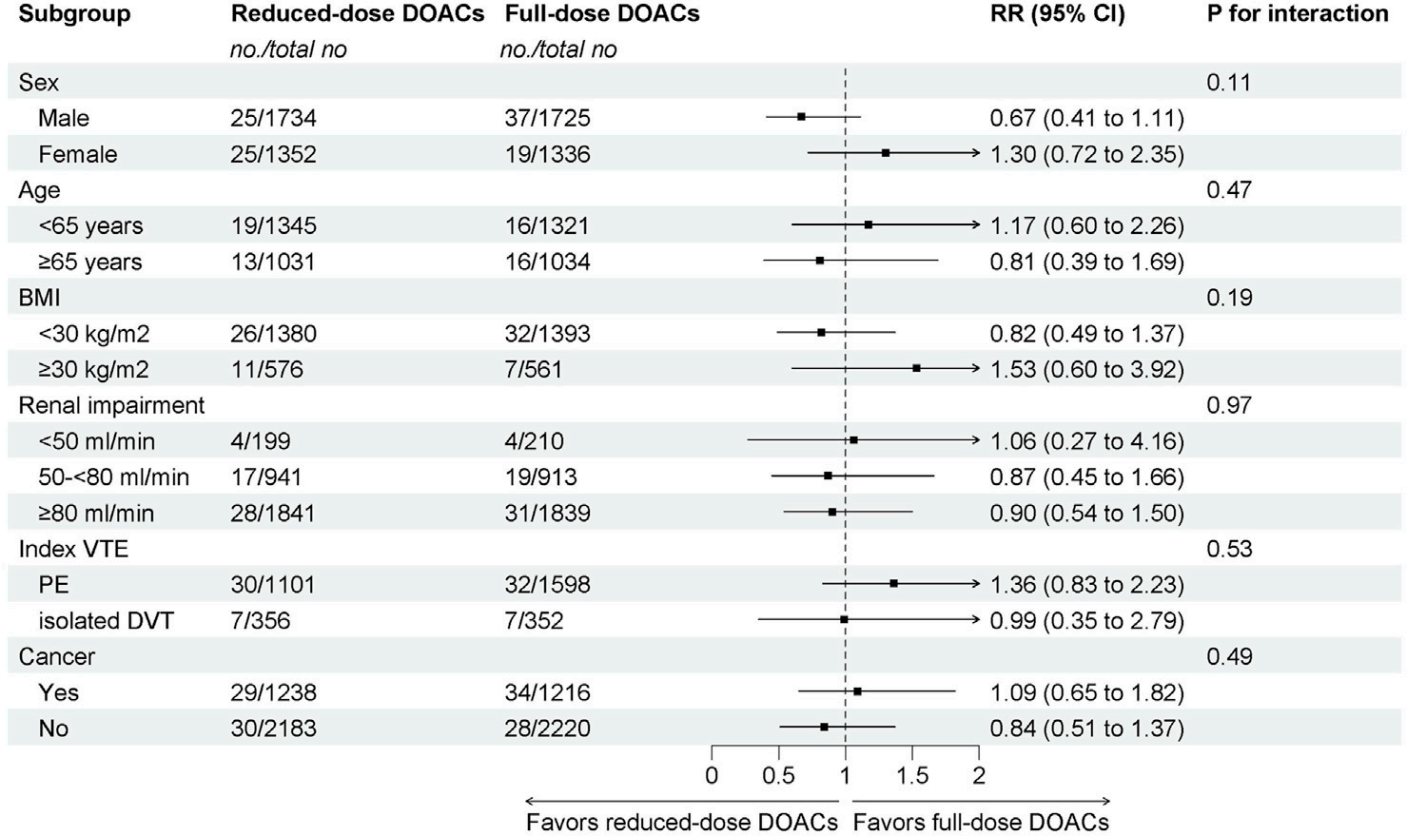
Subgroup analysis of Recurrent VTE.

The subgroup analyses demonstrated that reduced-dose DOACs did not significantly increase the risk of recurrent VTE compared to full-dose DOACs, regardless of sex, age, BMI, renal function, VTE type, or cancer. Test for subgroup differences indicated that there was no statistically significant subgroup effect (p = 0.11 for sex, p = 0.47 for age, p = 0.19 for BMI, p = 0.97 for renal impairment, p = 0.53 for index VTE, p = 0.49 for cancer). These results indicate that sex, age, BMI, renal function, VTE type, or cancer, as included in the trials, did not modify the effect of reduced-dose DOACs in comparison to full-dose DOACs on the risks of recurrent VTE (Figure 2).

### 3.2 Major bleeding/CRNMB

**FIGURE 3:**
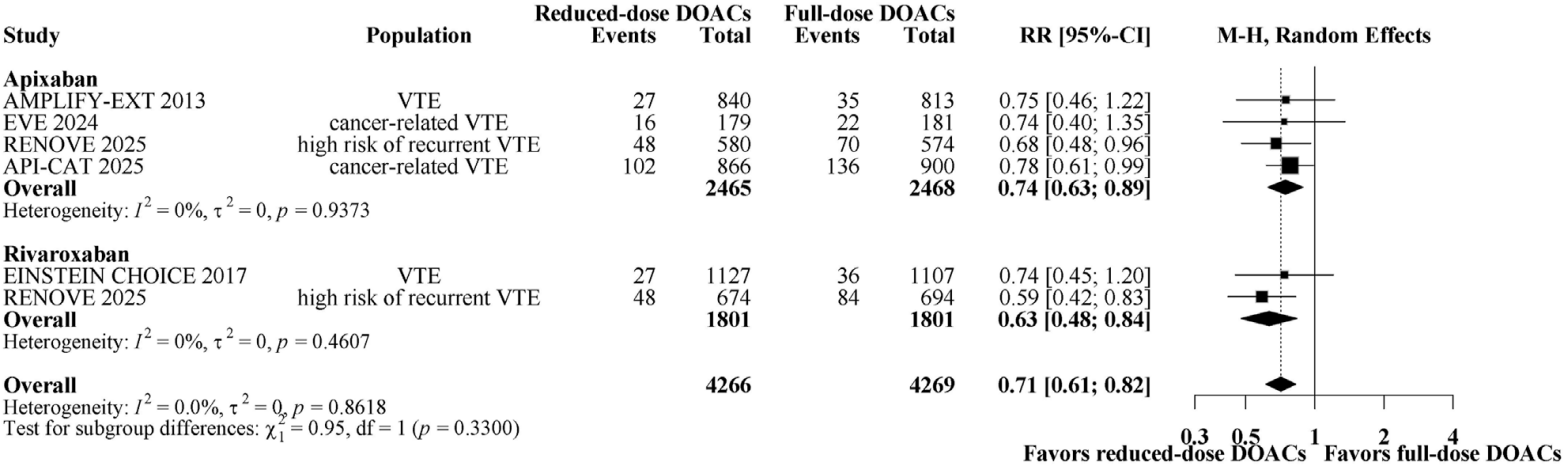
Forest plot of randomized controlled trials comparing reduced-dose DOACs vs. full-dose DOACs for risk for Major bleeding/CRNMB.

A total of 651 patients (7.63%) had major bleeding/CRNMB. Figure 3 shows a forest plot of comparing reduced-dose versus full-dose DOACs on risk for major bleeding/CRNMB. Reduced-dose DOACs was associated with significantly lower risk of major bleeding/CRNMB compared with full-dose DOACs (6.3% vs 9%; RR, 0.71; 95% CI, 0.61–0.82). There was no heterogeneity among DOACs (overall I^2^ = 0%). In the analysis by DOAC agents, the respective estimates for major bleeding/CRNMB risk were as follows: apixaban, RR, 0.74 (95% CI, 0.63–0.89); rivaroxaban, RR 0.63 (95% CI, 0.48–0.84). Sensitivity analysis indicated that the results remain unchanged when using a fixed-effect model (Supplementary Figure 5B). The quality of evidence was high for the comparison between reduced-dose and full-dose DOACs (Table 2). Major and CRNMB bleeding events were reported separately (Supplementary Figures S4, S5). Reduced-dose DOACs was associated with significantly lower risks of major bleeding or CRNMB compared with full-dose DOACs [(RR, 0.60; 95% CI, 0.41–0.88), (RR, 0.75; 95% CI, 0.63–0.88)].

**FIGURE 4:**
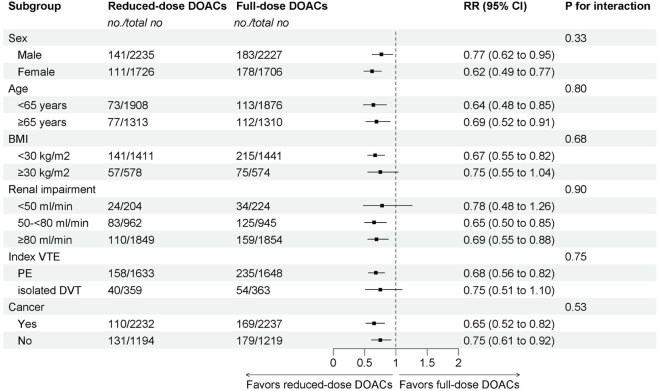
Subgroup analysis of Major bleeding/CRNMB.

The subgroup analyses demonstrated that reduced-dose DOACs did significantly decrease the risk of Major bleeding/CRNMB compared to full-dose DOACs, regardless of sex, age, BMI, renal function, VTE type, or cancer. Test for subgroup differences indicated that there was no statistically significant subgroup effect (p = 0.33 for sex, p = 0.80 for age, p = 0.68 for BMI, p = 0.90 for renal impairment, p = 0.75 for index VTE, p = 0.53 for cancer). These results indicate that sex, age, BMI, renal function, VTE type, or cancer, as included in the trials, did not modify the effect of reduced-dose DOACs in comparison to full-dose DOACs on the risks of Major bleeding/CRNMB (Figure 4).

### 3.3 Other results

**FIGURE 5:**
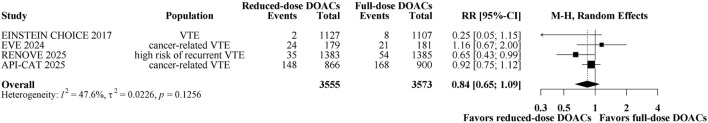
Forest plot of randomized controlled trials comparing reduced-dose DOACs vs. full-dose DOACs for risk for death.

A total of 460 patients (6.45%) had death. Figure 5 shows a forest plot of comparing reduced-dose versus full-dose DOACs on risk for death. Reduced-dose DOACs was not associated with significantly higher risks of death compared with full-dose DOACs (5.9% vs 7%; RR, 0.84; 95% CI, 0.65–1.09). There was no heterogeneity among studies (overall I^2^ = 47.65%). Sensitivity analysis indicated that the results remain unchanged when using the fixed-effect model (Supplementary Figure 5C). The quality of evidence was moderate for the comparison between reduced-dose and full-dose DOACs, also being graded down because of imprecision (Table 2).

## 4 Discussion

In this systematic review and meta-analysis of randomized controlled trials comparing reduced-dose with full-dose DOACs for the treatment of extended VTE (5 trials included for the primary outcome; 8,781 participants). We found no significant increase in risk of recurrent VTE, but significant decrease in risk of major bleeding/CRNMB. Additionally, our exploration of heterogeneity among trials suggested consistent effects by agent. Our subgroups results suggest a decreased risks of major bleeding/CRNMB, in patients taking reduced-dose DOACs compared to full-dose DOACs, regardless of sex, age, BMI, renal impairment, index VTE, and cancer.

These findings provide pooled estimates from high-quality studies to guide clinical decision-making. For patients requiring extended VTE therapy, there is currently insufficient strong evidence demonstrating that reduced-dose anticoagulation is superior to full-dose anticoagulation, resulting in some guidelines being unable to provide strong recommendations. Our results expand the existing knowledge on this topic by providing pooled estimates from five RCTs that increased statistical power and by providing subgroup-specific analyses. None of the tests for subgroup differences was statistically significant to indicate effect modification in such a way that reassures about the safety of reduced-dose DOACs for patients with extended VTE treatment. The difference in point estimates for major bleeding/CRNMB events among renal impairment could suggest an effect modification, such that even high risks of major bleeding/CRNMB events are observed for GFR <50 mL/min who receive reduced-dose DOACs compared to GFR ≥50–80 and ≥80 mL/min (RR: 0.65 vs 0.69 vs 0.78). Nonetheless, patients of both renal impairment manifested decreased risks of major bleeding/CRNMB on reduced-dose DOACs compared to full-dose DOACs.

Sex and age did not affect the superior safety profile of reduced-dose direct oral anticoagulants (DOACs) compared to the full-dose regimen, whereas the optimal dosing for other patient populations, such as those with cancer, obesity, renal impairment or isolated DVT, remains contested. Cancer-associated venous thromboembolism requires further prospective studies to determine whether it is beneficial to reduce the dose of DOACs during extended anticoagulation therapy. The EVE and API-CAT trials recently demonstrated that reduced-dose DOACs have more net clinical benefits than full-dose DOACs (McBane et al., 2024; Mahé et al., 2025). To further investigate this in the context of cancer, we performed a subgroup analysis incorporating data from the RENOVE trial, which specifically included high-risk patients with cancer-associated VTE (Couturaud et al., 2025). Our findings demonstrate that reduced-dose DOACs are indeed safer for managing cancer-related VTE.

Currently, there is still a lack of evidence regarding the applicability of reduced-dose DOACs in the extended VTE treatment of obese patients (Martin et al., 2021; Rosovsky et al., 2023). The previous EINSTEIN CHOICE and AMPLIFY EXTENSION trials had different weight groupings, making it impossible to conduct a meta-analysis to provide evidence-based guidance (Agnelli et al., 2013; Weitz et al., 2017). Therefore, we conducted a subgroup analysis based on BMI. The results showed that reduced-dose DOACs are suitable not only for patients with BMI <30 kg/m^2^ but also for those with BMI ≥30 kg/m^2^, thus filling this clinical gap. The routine use of reduced-dose DOACs provides greater protection for VTE patients with moderate to severe renal impairment, especially apixaban (Knueppel et al., 2022). However, the advantages of reduced-dose DOACs in the extended treatment of VTE associated with renal impairment remain insufficiently established. Our subgroup analysis based on renal impairment revealed that for patients with an estimated glomerular filtration rate (eGFR) <50 mL/min, reduced-dose DOACs are safer than full-dose DOACs. However, no significant difference was observed between full-dose and reduced-dose DOACs in patients with an eGFR ≥50 mL/min. This suggests that reduced-dose DOACs may offer additional advantages in the extended management of VTE associated with renal impairment.

Similarly, we conducted a subgroup analysis based on VTE classification for patients with independent DVT. The results showed no significant difference in recurrent VTE or massive bleeding/CRNMB events between DOACs with reduced and full doses. This indicates that reduced-dose DOACs can be used for extended therapy in patients with independent DVT. Through our subgroup analyses, we have further identified populations requiring reduced-dose DOACs during extended VTE treatment, such as those with cancer, obesity, renal impairment, and isolated DVT. However, more research is needed to refine dosing strategies for these specific groups.

This study has several limitations. First, our findings were imprecise due to the low incidence of certain outcomes and wide confidence intervals, especially for recurrent VTE. Second, there are differences in the enrolled populations across different trials (e.g., cancer patients and those with high-risk recurrent VTE). Although the absolute risk of bleeding events may vary by population, the absolute risk estimates of treatment effects are expected to be consistent across populations. Third, we did not find studies comparing doses of dabigatran and edoxaban, and it is unclear whether our results can be extrapolated to these scenarios. Fourth, given that only a few studies were identified for each comparison of reduced-dose DOACs versus full-dose DOACs, we did not create funnel plots to assess publication bias due to the increased risk for potential error (Sterne et al., 2011). This meta-analysis expands on previous work by including 5 large randomized trials and provides the most up-to-date synthesis of evidence synthesis to address this clinical question.

In summary, this study provides crucial evidence-based support for extended VTE therapy, demonstrating that reduced-dose DOACs significantly lowers the risk of major bleeding while maintaining anticoagulation efficacy. The consistent benefit-risk advantage demonstrated across key patient subgroups, including variations in sex, age, cancer, obesity, renal impairment, and isolated DVT, lays a robust foundation for treatment personalization. However, prospective studies are warranted to refine precise dosing strategies in these subgroups, to address the current evidence gaps for dabigatran and edoxaban, and to optimize the overall anticoagulation management strategy.

## 5 Conclusion

In conclusion, this meta-analysis founds that reduced-dose DOACs significantly lowered the risk of major bleeding/CRNMB compared to full-dose DOACs, with no significant change in the risk of recurrent VTE. Subgroup analyses based on sex, age, weight, renal impairment, type of VTE, and cancer did not show evidence of effect modification. This provides definitive evidence that reduced-dose DOACs should be considered the preferred strategy for most patients requiring extended VTE anticoagulation.

## Data Availability

The original contributions presented in the study are included in the article/Supplementary Material, further inquiries can be directed to the corresponding author.
