# Supplementary material for: Full-dose versus reduced-dose comparison of direct oral anticoagulants for extended treatment of venous thromboembolism: a systematic review and meta-analysis of randomized controlled trials
Source: Front Pharmacol. 2025 Dec 4;16:1708316. doi: 10.3389/fphar.2025.1708316 (PMC12711545; doi:10.3389/fphar.2025.1708316)
Supplement: Supplementary file 1 [file Supplementaryfile1.docx]

**Supplemental material**

[Supplementary methods. Search strategy 2](#_Toc212746510)

[Supplementary Figure 1. PRISMA Diagram 3](#_Toc212746511)

[Supplementary Figure 2. Risk of bias assessment for included trials by use of the Cochrane tool. + represents low risk of bias. 4](#_Toc212746512)

[Supplementary Figure 3. Forest plot of randomized controlled trials comparing reduced-dose DOACs vs. full-dose DOACs for risk for Major bleeding. 5](#_Toc212746513)

[Supplementary Figure 4. Forest plot of randomized controlled trials comparing reduced-dose DOACs vs. full-dose DOACs for risk for CRNMB. 5](#_Toc212746514)

[Supplementary Figure 5. Sensitivity analysis using fixed effect and risk ratio 6](#_Toc212746515)

# [Supplementary](https://journals.sagepub.com/doi/suppl/10.1177/10760296251372947) methods. Search strategy

Studies were searched through June 30th 2025.

Search terms were as follows:

(("Venous Thromboembolism"[Mesh] OR "Venous Thrombosis"[Mesh] OR "Pulmonary Embolism"[Mesh]) OR ("venous thromboembolism" OR "VTE" OR "deep vein thrombosis" OR "DVT" OR "pulmonary embolism" OR "PE"))

AND (("Anticoagulants"[Mesh] OR "Factor Xa Inhibitors"[Mesh]) OR ("direct oral anticoagulant" OR "DOAC" OR "NOAC" OR "factor Xa inhibitor" OR "direct thrombin inhibitor" OR "dabigatran" OR "rivaroxaban" OR "apixaban" OR "edoxaban"))

AND (("Dose-Response Relationship, Drug"[Mesh] OR "Drug Administration Schedule"[Mesh]) OR ("full dose" OR "standard dose" OR "therapeutic dose" OR "reduced dose" OR "low dose" OR "prophylactic dose" OR "extended treatment" OR "secondary prevention" OR "long-term anticoagulation"))

AND (("Randomized Controlled Trial"[Publication Type] OR "randomized controlled trial" OR "RCT" OR "randomised controlled trial" OR "clinical trial" OR "controlled clinical trial"))

The search was conducted through PubMed.

# [Supplementary](https://journals.sagepub.com/doi/suppl/10.1177/10760296251372947) Figure 1. PRISMA Diagram

**
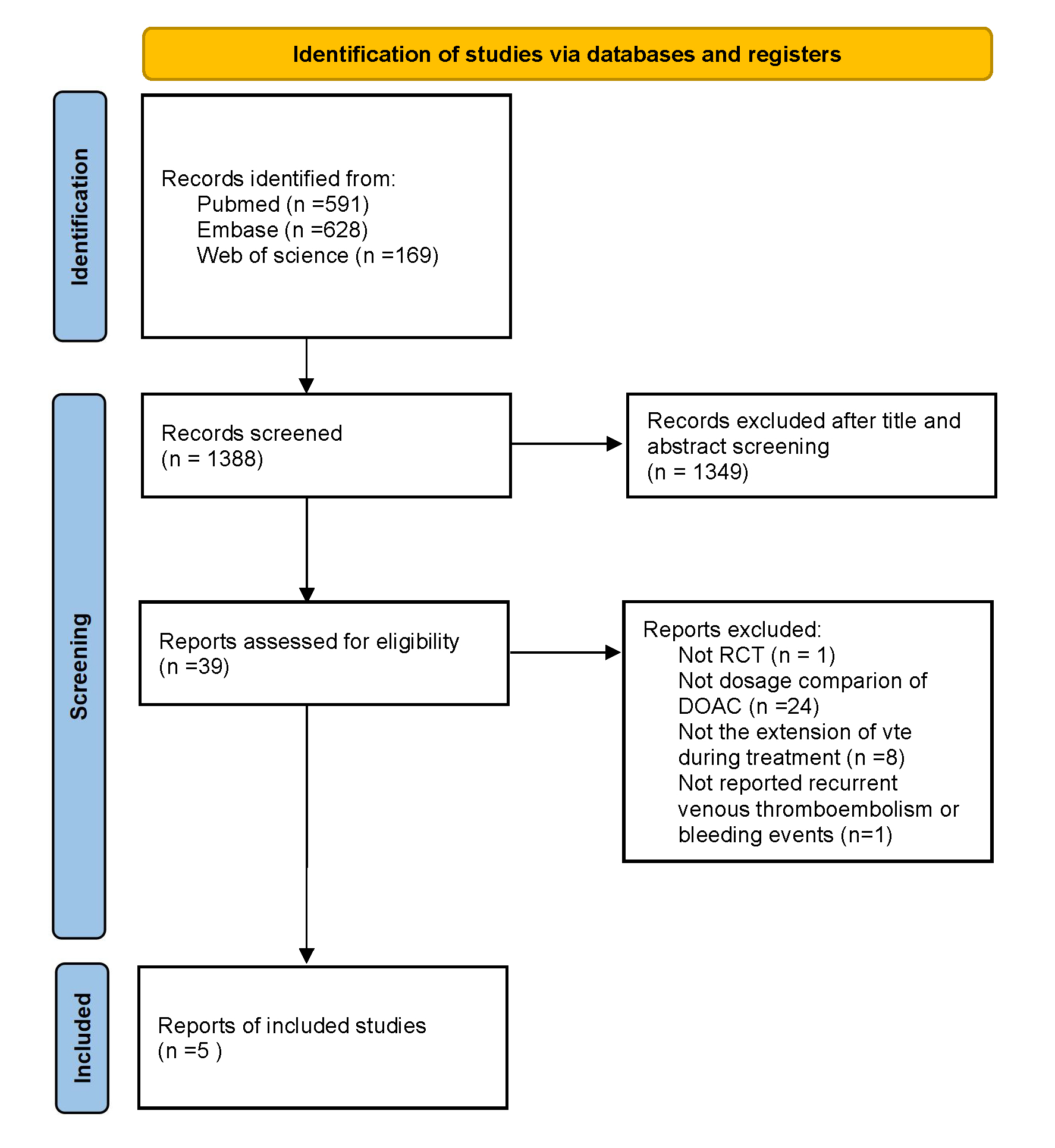
**

# [Supplementary](https://journals.sagepub.com/doi/suppl/10.1177/10760296251372947) Figure 2. Risk of bias assessment for included trials by use of the Cochrane tool. + represents low risk of bias.


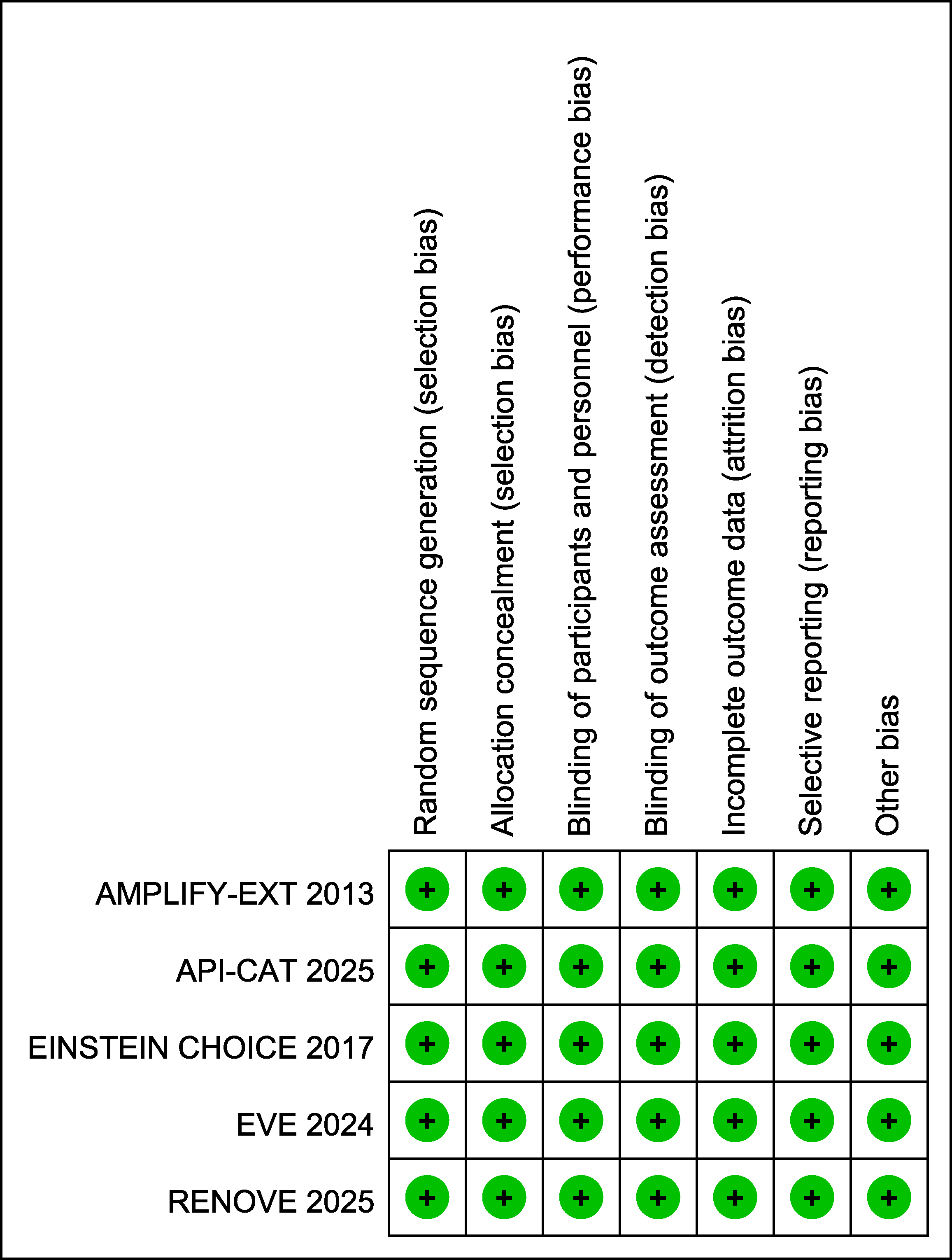


# [Supplementary](https://journals.sagepub.com/doi/suppl/10.1177/10760296251372947) Figure 3. Forest plot of randomized controlled trials comparing reduced-dose DOACs vs. full-dose DOACs for risk for Major bleeding.

**
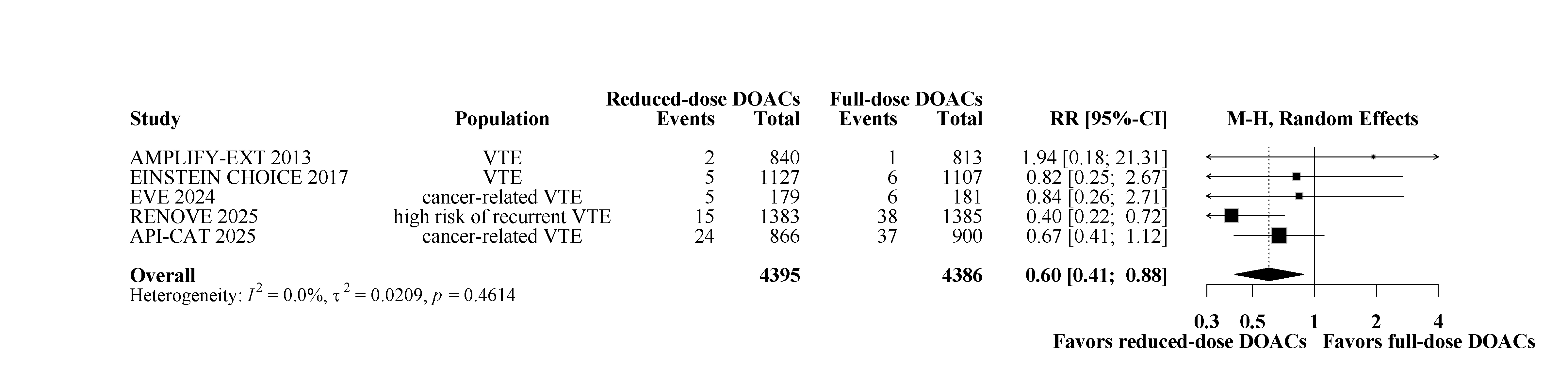
**

# [Supplementary](https://journals.sagepub.com/doi/suppl/10.1177/10760296251372947) Figure 4. Forest plot of randomized controlled trials comparing reduced-dose DOACs vs. full-dose DOACs for risk for CRNMB.


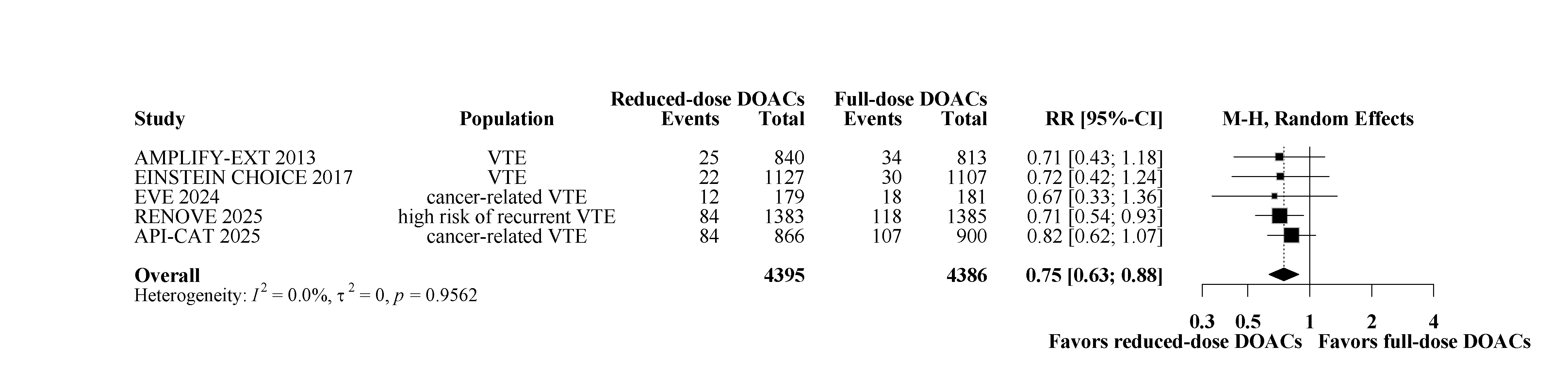


# [Supplementary](https://journals.sagepub.com/doi/suppl/10.1177/10760296251372947) Figure 5. Sensitivity analysis using fixed effect and risk ratio


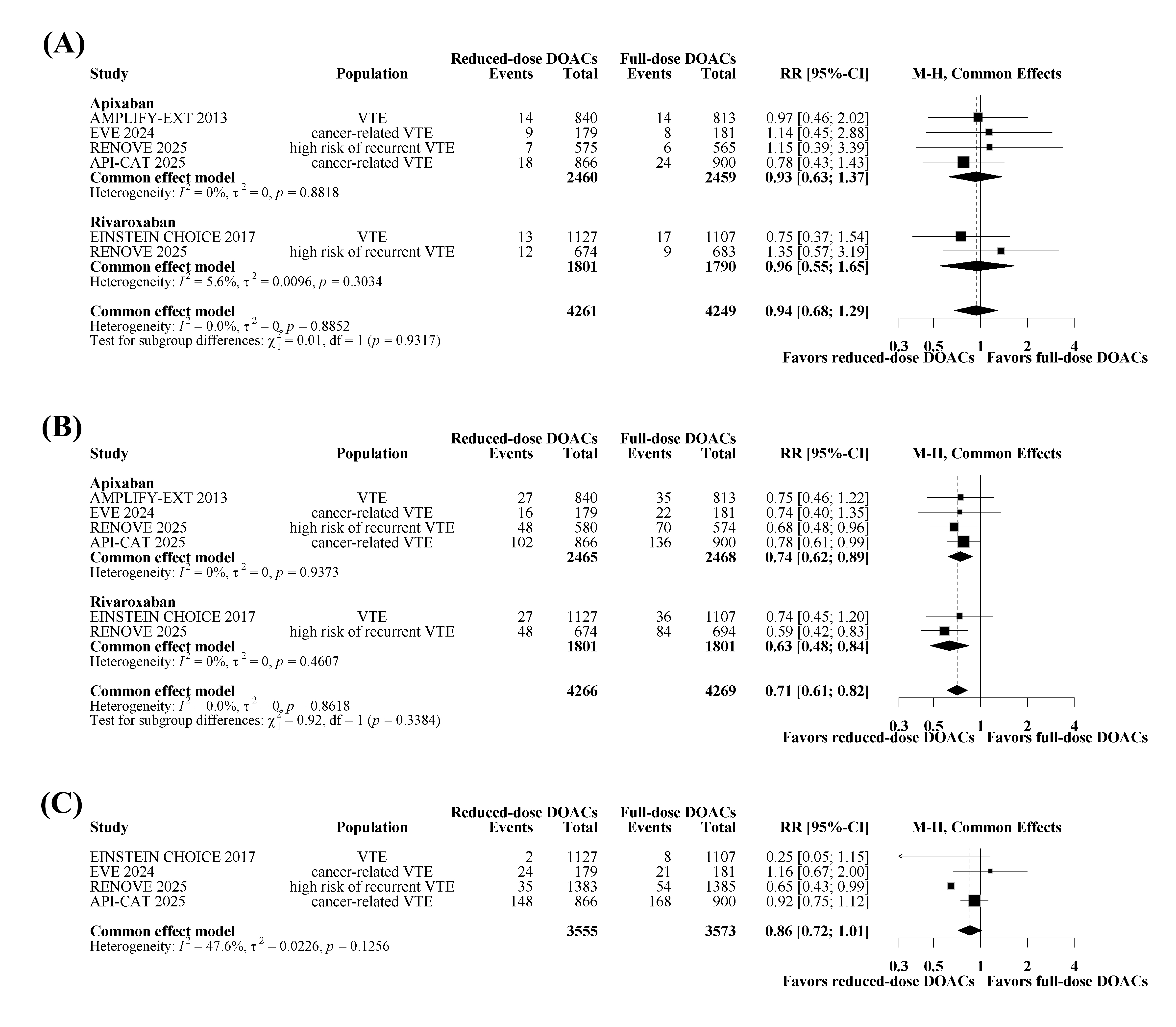


1. Forest plot of randomized controlled trials comparing reduced-dose DOACs vs.full-dose DOACs for risk for Recurrent VTE; B. Forest plot of randomized controlled trials comparing reduced-dose DOACs vs.full-dose DOACs for risk for Major bleeding/CRNMB; C. Forest plot of randomized controlled trials comparing reduced-dose DOACs vs.full-dose DOACs for risk for Death
